# Porin‐Independent Uptake of Small Molecule Antibiotics Facilitated by *Escherichia coli* Outer Membrane Vesicles

**DOI:** 10.1002/bit.70078

**Published:** 2025-10-03

**Authors:** Meishan Wu, Rachael M. Harrower, Ziang Li, Angela C. Brown

**Affiliations:** ^1^ Department of Chemical and Biomolecular Engineering Lehigh University Bethlehem Pennsylvania USA; ^2^ Department of Biological Sciences Lehigh University Bethlehem Pennsylvania USA

**Keywords:** antibiotics, imipenem, outer membrane vesicle, porins

## Abstract

The development of novel antimicrobial agents that are effective against Gram‐negative bacteria is hindered by the dual membrane cell envelope of these bacteria. To reach their intracellular targets, most small‐molecule antibiotics must first pass through protein channels called porins; however, a common mechanism of acquired resistance is decreased expression of these outer membrane proteins. Additionally, parameters such as size, shape, and charge regulate passage of antibiotics through porins, further limiting the design space of novel antibiotic molecules. Inspired by the ability of bacterial outer membrane vesicles (OMVs) to deliver cargo to the bacterial cytosol, we hypothesized that encapsulation of small molecule antibiotics within OMVs would improve the activity of the drugs by facilitating uptake. To test this hypothesis, we investigated the ability of imipenem‐encapsulated OMVs to inhibit the growth of several Gram‐negative bacteria, including multidrug‐resistant (MDR) clinical isolates. Our results demonstrated that encapsulation within OMVs significantly lowers the effective concentration of imipenem in several MDR isolates. Using a panel of porin knockout strains, we further demonstrated that this mechanism of antibiotic delivery does not require porin expression. Together, our results demonstrate the potential of OMVs as novel antibiotic delivery vehicles to treat antibiotic‐resistant bacterial infections by improving drug uptake.

## Graphical abstract

We demonstrate that bacterial outer membrane vesicles (OMVs) can enhance antibiotic delivery into Gram‐negative bacteria by bypassing porin‐dependent uptake. Encapsulation of imipenem in OMVs significantly increased its effectiveness against multidrug‐resistant clinical isolates, highlighting OMVs as a promising platform for overcoming permeability barriers in the treatment of resistant bacterial infections.

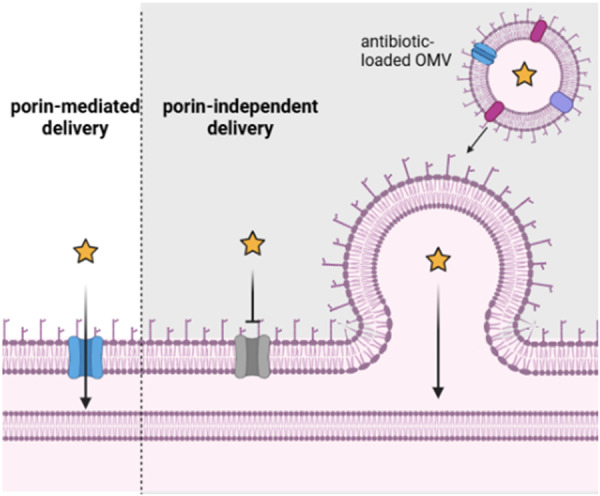

## 1 Introduction

The emergence and rapid spread of antibiotic resistance presents an alarming threat to global health. Among the list of 18 antibiotic‐resistant bacteria and fungi identified by the United States Centers for Disease Control and Prevention, 11 are Gram‐negative bacteria (CDC 2019). These pathogens exhibit resistance to a wide range of current antibiotics, leaving few therapeutic options. At the same time, development of new drugs that are effective against Gram‐negative bacteria has been limited; in fact, the last class of broad‐spectrum antibiotics to be developed was the quinolone family, which was first introduced in the 1960s. This lack of new drugs has led to a critical shortage of viable treatment options for infections caused by these resistant strains, with clinicians relying on older drugs such as colistin, despite their severe side effects and limited effectiveness (Boucher et al. 2009).

The primary factor leading to limited activity of many antibiotics against Gram‐negative bacteria is their impenetrable cell envelope, which is composed of an outer membrane and an inner membrane, separated by a periplasmic space. The outer membrane is rich in lipopolysaccharide (LPS), which serves as a protective barrier against harmful substances, such as antibiotics. Beneath the outer membrane lies the periplasmic space, filled with a gel‐like matrix containing a peptidoglycan layer that provides structural support to the cell. The inner membrane is composed of a phospholipid bilayer that regulates the transport of ions and molecules, further limiting uptake of antibiotics (Beveridge 1999). To reach their cytosolic targets, antibiotics must therefore pass through protein channels called porins. However, large antibiotics, such as glycopeptides are unable to pass through these porins (Delcour 2009), and bacteria can downregulate porin expression to limit drug uptake (Pagès et al. 2008).

Recent studies have therefore focused on enhancing antibiotic effectiveness by combining antibiotics with certain adjuvants to promote rapid drug uptake using membrane permeabilizers or lipid‐based nanodelivery platforms. Membrane permeabilizers, such as polymyxin B and ethylenediaminetetraacetic acid (EDTA), work by disrupting the structure of the bacterial outer membrane (Mohapatra et al. 2021; Prachayasittikul et al. 2007). When used in combination with antibiotics, membrane permeabilizers improve the transport of antibiotics across the outer membrane by increasing the fluidity of the lipid bilayer (Vaara 1992). However, the use of permeabilizers in vivo is limited by their adverse effects on mammalian cells, as the molecules can also affect the host cell membranes (Heindorff et al. 1983; Li et al. 2017). An alternative approach to improve antibiotic delivery involves the use of lipid‐based delivery systems, such as liposomes. These carriers are designed with specific lipid compositions that enable them to fuse with the bacterial outer membrane. Such fusogenic liposomes have been successfully employed to broaden the spectrum of antibiotics, such as vancomycin, which is otherwise ineffective against Gram‐negative bacteria (Nicolosi et al. 2015). Despite their promise, these lipid‐based nanodelivery systems face challenges related to their physical instability, which can lead to drug leakage over time (Liu et al. 2022).

Gram‐negative bacteria release vesicles derived from their outer membrane, called “outer membrane vesicles” (OMVs) throughout all stages of growth (Schwechheimer and Kuehn 2015). OMVs are capable of encapsulating and delivering nucleic acids, signaling molecules, enzymes, and toxins to promote the survival of parent bacteria (Brameyer et al. 2018; Chatterjee and Chaudhuri 2011; Ciofu 2000; Dauros‐Singorenko et al. 2018; Renelli et al. 2004). Importantly, these vesicles enable content delivery to bacterial cells by fusing with their outer membrane (Kadurugamuwa and Beveridge 1999) allowing the transfer of genetic material or other macromolecules directly into the recipient cells (S. Chatterjee et al. 2017; Lee et al. 2022; Rumbo et al. 2011; Shen et al. 2024). In addition, the stability of OMVs ensures the protection of their cargo in harsh environments (Alves et al. 2016).

We therefore proposed that OMVs might promote antibiotic delivery across the Gram‐negative cell membrane, thereby enhancing the effectiveness of these drugs (Collins and Brown 2021). We have previously explored several methods for the loading of fluoroquinolones into the lumen of *Escherichia coli* OMVs. Our results demonstrated that (1) small molecules can be readily loaded into *E. coli* OMVs, and (2) active loading methods, such as electroporation and sonication, can efficiently load antibiotics into OMVs with an encapsulation efficiency reaching as high as 68% (Wu et al. 2024).

In this study, we explored the antibacterial potential of antibiotic‐loaded OMVs (aOMVs) against laboratory and multidrug‐resistant (MDR) clinical isolates of Gram‐negative bacteria by comparing the activity of aOMVs against that of free (unencapsulated) antibiotics. To demonstrate that OMVs enable delivery via a porin‐independent pathway, we tested aOMVs on porin‐deficient single‐gene knockout mutants of *E. coli*. Together, our results demonstrate that by improving delivery across the Gram‐negative outer membrane, in a porin‐independent manner, OMVs are able to enhance the activity of small molecule antibiotics against a range of bacterial species, including MDR isolates.

## 2 Experimental

### 2.1 Bacterial Growth Conditions

The list of bacteria used in these experiments is included in Table 1. All *Pseudomonas aeruginosa* strains and *E. coli* strains, except for the Keio strains, were grown in Luria‐Bertani (LB) broth Lennox from Invitrogen (Waltham, MA). The *E. coli* Keio strains, including the wild type and mutants, were cultivated in LB low salt broth, supplemented with 25 µg/mL of kanamycin.

**Table 1:** List of bacteria used in this study.

*Species*	*Strain*	*Notable characteristics*	*Reference*
*E. coli*	W3110	K‐12 wild type	Bachmann (1972)
	JC8031	K‐12 mutant, Δ*tolRA*, hypervesiculating	Bernadac et al. (1998)
	BW25113	Keio collection wild type	Baba et al. (2006)
	JW0912	Keio mutant, Δ*ompF*	Baba et al. (2006)
	JW2203	Keio mutant, Δ*ompC*	Baba et al. (2006)
	JW0940	Keio mutant, Δ*ompA*	Baba et al. (2006)
	JW3368	Keio mutant, Δ*ompR*	Baba et al. (2006)
*P. aeruginosa*	PAO1	Wild type	Holloway (1955)
	PA0238	Clinical isolate from CDC	CDC (2024)
	PA0252	Clinical isolate from CDC	CDC (2024)
	PA0260	Clinical isolate from CDC	CDC (2024)

### 2.2 Chemicals

Imipenem (IMI) was purchased from Fisher Scientific (Waltham, MA). All chemicals were used without any additional purification.

### 2.3 Isolation and Purification of OMVs

A starter culture of *E. coli* JC8031 was made by combining 10 mL LB Lennox with 100 µL of a glycerol stock of the bacterium; this culture was grown at 37°C with shaking at 175 RPM for 8 to 12 h. The starter culture was further diluted with LB at a 1:100 ratio and grown until the culture reached the late exponential phase. The supernatant was separated from the bacteria by centrifugation at 10,000 × g for 10 min at 4°C, followed by a second centrifugation at 10,000 x g for 5 min at 4°C in a new container. The supernatant was then filtered through a 0.45 µm polyethersulfone (PES) membrane to remove any remaining cells. Before ultracentrifugation, the supernatant was concentrated to approximately 150 mL using centrifugal concentrator tubes with a 50 kDa molecular weight cut‐off (MWCO), by centrifuging at 5,000 x g for 10 min at 4°C. The concentrated supernatant was then ultracentrifuged twice at 175,000 x g for 1 h at 4°C. The pellet was resuspended in 1 mL phosphate buffered saline (PBS) and filtered through a 0.45 µm PES syringe filter before being stored at −20°C for further use.

### 2.4 Synthesis of aOMVs

Antibiotics were loaded into OMVs using sonication following a published protocol (Lamichhane et al. 2016; Wu et al. 2024). The OMVs were mixed with an equal volume of a 0.1% w/v antibiotic solution. The mixture was incubated at room temperature for 30 min, then sonicated in a bath sonicator (VWR) for 30 s at 35 kHz, then placed on ice for 60 s, followed by another sonication in the bath sonicator for 30 s at the same settings. The sonicated mixture was incubated at room temperature for 1 h to allow OMV recovery. After loading was completed, the OMV‐antibiotic mixture was transferred to an Amicon Ultra 30‐kDa MWCO centrifugal filter and centrifuged at 14,000 x g for 15 min at 4°C.

The filtrate, which contained unencapsulated antibiotics, was collected and diluted with PBS. The absorbance of each dilution was measured using a Tecan Infinite® 200 PRO plate reader. The encapsulation efficiency (EE) was calculated using Equation 1, where $a_{\mathrm{total}}$is the total mass of antibiotic added into the mixture; and $a_{\mathrm{free}}$ is the mass of unencapsulated antibiotic, which was determined by comparing the absorbance of the filtrate with a calibration curve (Figure S1).

(1) $$\mathrm{EE}=\frac{\left(a_{\mathrm{total}}-a_{\mathrm{free}}\right)}{a_{\mathrm{total}}}*100\;\%$$

### 2.5 Measurement of Vesicle Size

The diameter of the OMVs was measured using dynamic light scattering (DLS). The sample was diluted with PBS at a 1:20 volume ratio and filtered through a PES membrane with 0.45 µm pore size to eliminate any potential contamination. We used an ALV/CGS‐3 compact goniometer system, measured at a wavelength of 632.8 nm and scattering angle of 90°, with a run duration of 180 s. The data were collected in triplicate. The ALV built‐in software was used for data processing. The number‐weighted size distribution was plotted with the ALV built‐in software program using a regularized fit and a membrane thickness of 5 nm (r* = 5 nm).

### 2.6 Determination of Lipid Content

The lipid content of the OMVs was determined using the lipophilic FM^TM^ 4‐64 dye (Invitrogen). Liposomes composed of 1‐palmitoyl‐2‐oleoylglycero‐3‐phosphocholine (POPC) at known lipid concentrations were used as a comparison. After incubating 200 ng of the dye per 50 µL of diluted sample in the dark for 15 s, the fluorescence spectra were recorded with an excitation wavelength of 515 nm and emission wavelength of 640 nm using a PTI QuantaMaster fluorometer.

### 2.7 Scanning Electron Microscopy Analysis

Scanning electron microscopy (SEM) was used to visualize the integrity and size of the OMVs. The images were collected using a Hitachi Scientific Instrument S4300SE Schottky‐Emission SEM. The samples were first fixed using 2% glutaraldehyde for 2 h in microcentrifuge tubes, then rapidly frozen in liquid nitrogen before being lyophilized. Following the deposition of lyophilized OMVs on aluminum stubs, the samples were coated with iridium using an EMS575X sputter coater, with a thickness of approximately 3 nm. Visualization was achieved using an accelerating voltage of 5 kV.

### 2.8 aOMV Effectiveness Assay

The effectiveness of the aOMVs was analyzed by assessing their ability to inhibit bacterial growth over a range of antibiotic concentrations. The bacteria were treated with (1) PBS, (2) free antibiotics in a range of concentrations, (3) aOMVs with the same antibiotic concentrations as treatment 2, or (4) empty OMVs with the same lipid concentration as treatment 3. Bacterial growth at 37°C was monitored by collecting hourly readings of the optical density at 600 nm (OD_600_) using a Tecan Infinite® 200 PRO plate reader. All samples were prepared in triplicate in a sterile 96 well‐plate. The data were normalized using the growth of untreated bacteria to compare the change in bacterial growth for each treatment.

For each set of samples in which an improvement was observed in the aOMVs relative to the free IMI, a percent improvement was calculated:

(2) $$\%\text{improvement}=\frac{{\text{OD}}_{600}\left(\text{free IMI}\right)-{\text{OD}}_{600}\left(\text{IMI}-\text{OMVs}\right)}{{\text{OD}}_{600}\left(\text{free IMI}\right)}$$

where OD_600_(free IMI) represents the OD_600_ after treatment with free IMI, and OD_600_(IMI‐OMVs) represents the OD_600_ after treatment with IMI‐OMVs.

### 2.9 aOMV Leakage

The stability of the aOMVs was assessed by determining the extent of leakage of encapsulated content under various conditions. aOMVs with known concentrations of encapsulated antibiotic were stored in a 37°C incubator for 24 h. PBS was added to rinse any potential antibiotic from the aOMV surface and collect drug molecules leaked from the interior of aOMVs. The mixture was transferred to a concentrator with 30 kDa MWCO and centrifuged at 14,000 x g for 15 m to separate the leaked antibiotic and membrane fragments from intact aOMVs. The UV‐Vis absorbance of the filtrate was compared against a calibration curve to calculate the concentration of released content.

## 3 Results

### 3.1 Characterization and Stability of aOMVs

**Figure 1:**
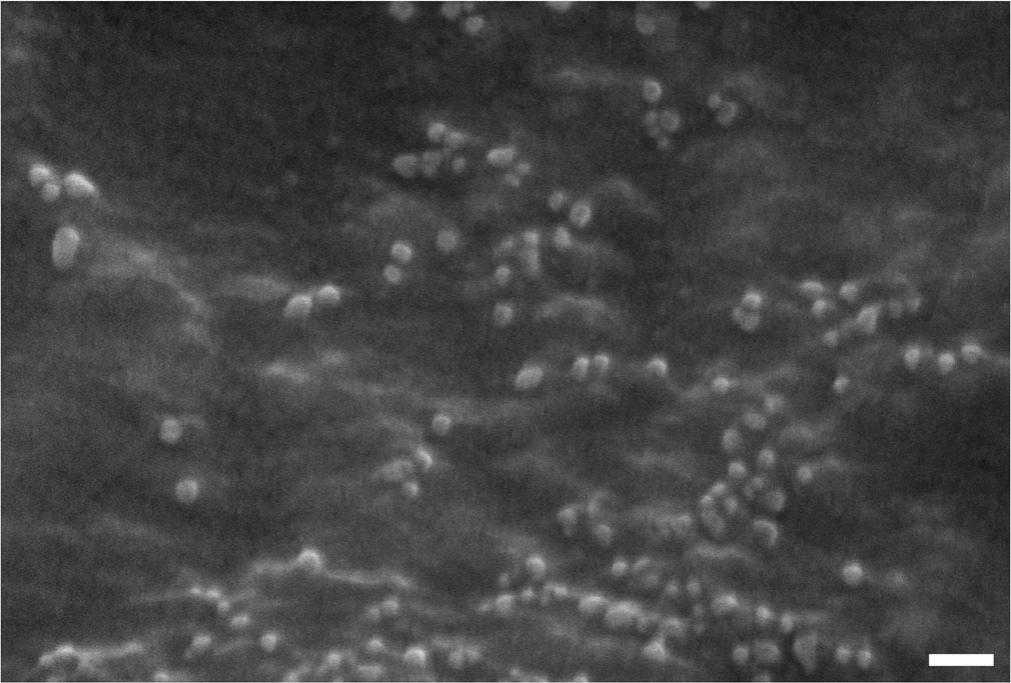
*E.coli* JC8031 OMVs imaged using SEM. Unloaded OMVs are spherical and homogeneous in size with an average diameter of 33.7 ± 7.0 nm (*n* = 25) analyzed using ImageJ. Scale bar = 100 nm.

The morphology of the empty OMVs, determined using SEM, is displayed in Figure 1. The empty OMVs appeared spherical, with an average diameter of 33.7 ± 7.0 nm (*n* = 25). The sizes of the OMVs and aOMVs measured using DLS are reported in Table 2. The measured diameter of the untreated OMVs is consistent with those observed using SEM. No significant difference in size was observed between the unloaded OMVs and those loaded with IMI.

**Table 2:** Average diameter of OMVs and aOMVs measured using DLS.

*Condition*		*Diameter (nm)*
Untreated	Empty OMVs	38.33 ± 3.64
	IMI‐OMVs	40.38 ± 3.29
Post‐incubation (24 h, 37°C)	Empty OMVs	42.98 ± 2.33
	IMI‐OMVs	39.64 ± 1.46

*Note:* The diameter represents the mean (*n* = 3) ± standard deviation.

Sonication was used to synthesize aOMVs, and the encapsulation efficiency (EE) for IMI‐loaded OMVs (IMI‐OMVs) was found to be 41% ± 2.4%. We also investigated OMV stability by incubating the aOMVs at 37°C for 24 h. We showed, using DLS, that the size of the OMVs did not change upon incubation for 24 h (Table 1); in addition, the content leakage from IMI‐OMVs was determined to be less than 1% of the total amount encapsulated. These results indicate that OMVs are resistant to thermal degradation and can provide a stable environment for encapsulated cargo.

### 3.2 Delivery of aOMVs to Gram‐Negative Bacteria

**Figure 2:**
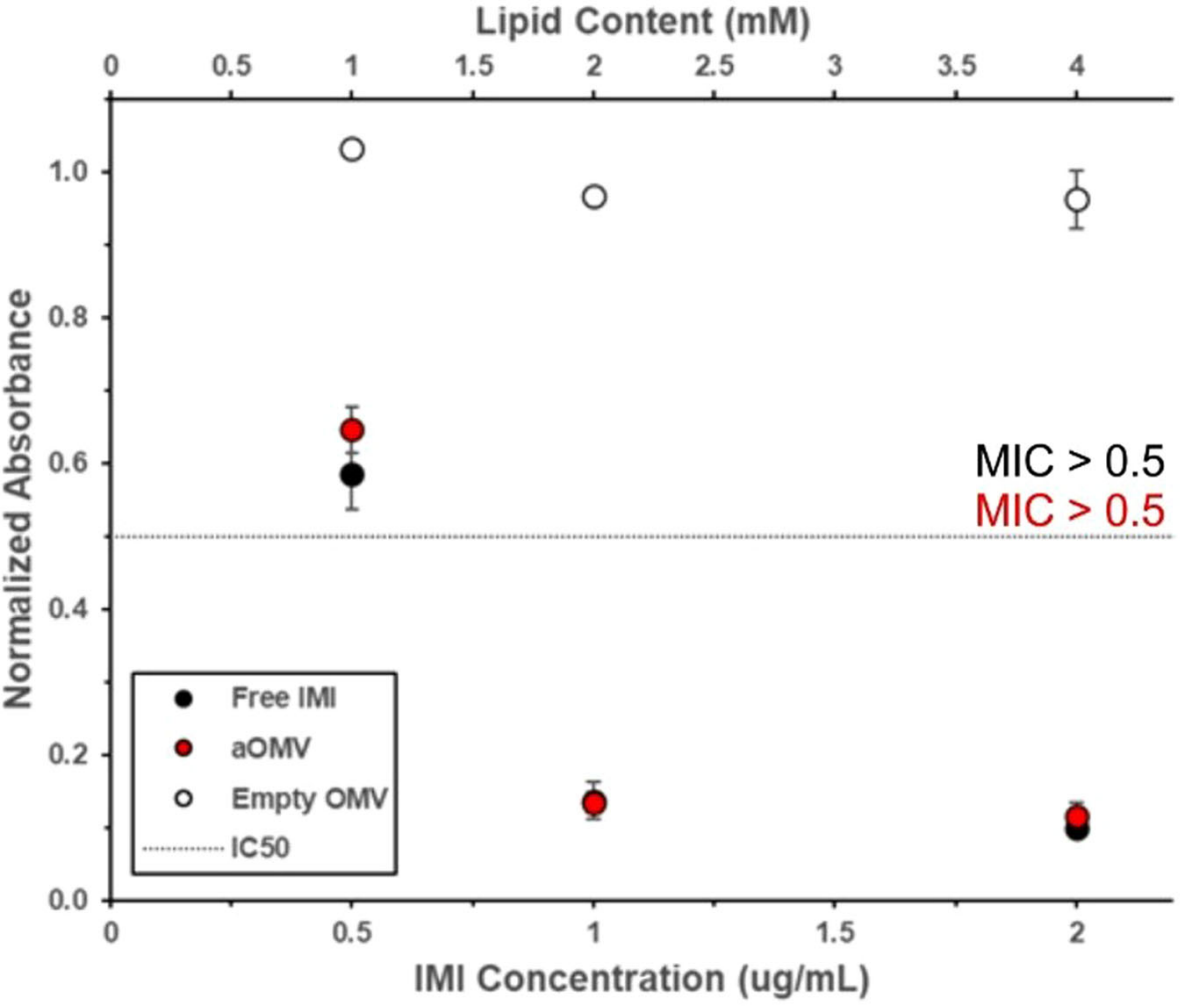
Delivery of IMI, both free and encapsulated within OMVs, to *P. aeruginosa* strain PAO1. Bacteria were treated with free IMI (black), IMI‐OMVs (red), or empty OMVs (white). The change in absorbance at 600 nm after 12 h of growth of treated bacteria was normalized to that of untreated bacteria. The minimum inhibitory concentration (MIC) for both free IMI and IMI‐OMVs is greater than 0.5 µg/mL. Each data point represents the mean (*n* = 3) ± standard deviation. The dashed line represents the IC_50_.

We previously showed that encapsulation of IMI in OMVs enhances the antibacterial activity of this drug against a lab strain of *E. coli*, W3110 (Wu et al. 2024). To demonstrate the wider applicability of the approach, we tested the effectiveness of IMI‐OMVs against a lab strain of *P. aeruginosa*, PAO1. PAO1 cells were treated with free IMI, IMI‐OMVs (with the same IMI concentration) or empty OMVs (with the same OMV concentration). Bacterial growth was determined by following the OD_600_ over time. The half‐maximal normalized absorbance of the PBS‐treated control (IC_50_), served as a reference, where values below the IC_50_ were determined to represent “effective” concentrations. We observed that IMI‐OMVs were as effective as free IMI in inhibiting the growth of this strain of bacteria (Figures 2 and S2, Table S1), with both IMI and IMI‐OMVs crossing the IC_50_ at a concentration of 1 µg/mL. Empty OMVs had limited effect on bacterial growth.

**Figure 3:**
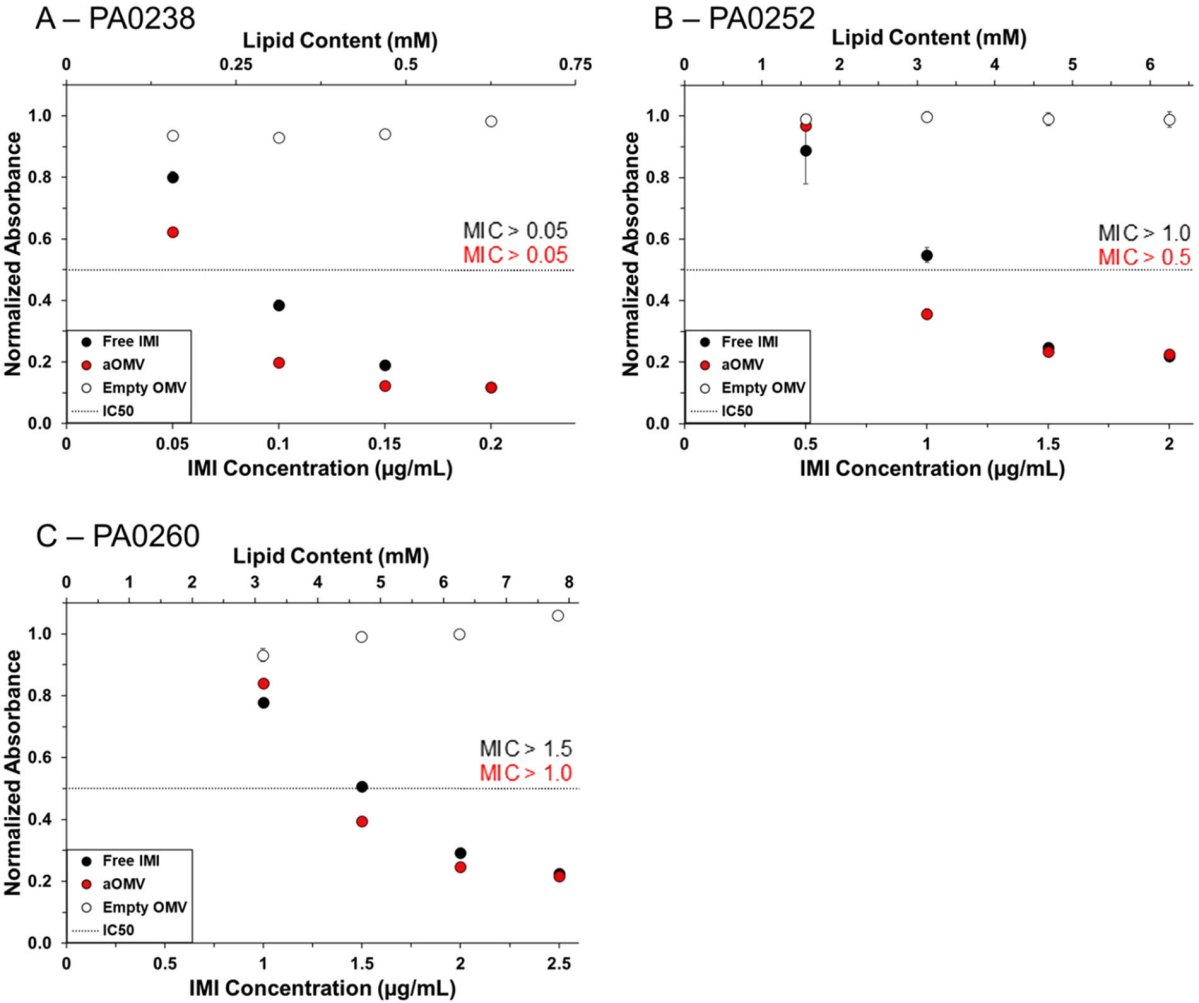
Delivery of IMI, both free and encapsulated within OMVs, to MDR *P. aeruginosa* clinical isolates (A) PA0238, (B) PA0252, and (C) PA0260. Bacteria were treated with free IMI (black), IMI‐OMVs (red), or empty OMVs (white). The change in absorbance at 600 nm after 12 h of growth of treated bacteria was normalized to that of untreated bacteria. The minimum inhibitory concentration (MIC) for free IMI and IMI‐OMVs is marked in black and red, respectively. Each data point represents the mean (*n* = 3) ± standard deviation. The dashed line represents the IC_50_.

Next, we furthered these studies by investigating the activity of aOMVs in MDR clinical isolates of *P. aeruginosa*, obtained from the CDC/FDA Antimicrobial Resistance Isolate Bank. Figure 3 shows the change in absorbance after 12 h for bacteria treated with either free IMI, IMI‐OMVs, or empty OMVs, normalized by the change in absorbance of untreated bacteria.

Isolate PA0238 was highly susceptible to IMI, as demonstrated by the IC_50_ of free IMI of 0.1 µg/mL (Figures 3A and S3, Table S2). IMI‐OMVs, with an IMI concentration of 0.1 µg/mL, were even more effective in inhibiting bacterial growth than was the free drug at similar drug concentrations. Empty OMVs at the same concentration had no effect on PA0238 growth, indicating that the enhanced effectiveness of IMI‐OMVs was not due to any component of the OMVs themselves. Isolates PA0252 and PA0260 were less susceptible to IMI, with IC_50_ values of 1.5 and 2 µg/mL, respectively. IMI‐OMVs similarly improved the effectiveness of IMI in both isolates, lowering the IC_50_ values to 1.0 and 1.5 µg/mL respectively (Figures 3B,C and S3, Table S2). Again, these differences were not due to any components of the OMVs, as empty OMVs at the same lipid concentration did not inhibit bacterial growth. These results demonstrate that aOMVs can lower the inhibitory antibiotic concentration relative to free antibiotics in bacteria that are fully or moderately susceptible to the drug.

### 3.3 Mechanism of aOMV Delivery

To determine whether aOMVs enable transport of antibiotics in a porin‐independent manner as we hypothesized, we compared the growth of wild type *E. coli* K‐12 BW25113, against several mutants from the Keio collection, including JW0912 (∆*ompF*), JW2203 (∆*ompC*), JW0940 (∆*ompA*), and JW3368 (∆*ompR*), each carrying a single‐gene deletion (Baba et al. 2006). OmpF is an essential pathway for the transport of small antibiotic drug molecules, including IMI across the membrane (Masi and Pagès 2013). OmpA, on the other hand, has a low permeability for small ions and is mainly associated with membrane structural integrity (Sugawara and Nikaido 1992). OmpC has a role in both, maintaining structural integrity while allowing the passage of small molecules (Masi and Pagès 2013). OmpR is a transcription factor that regulates the expression of porin genes in response to changes in the environmental osmotic pressure (Gerken et al. 2020).

As demonstrated by the experimental IC_50_ of IMI against the parent strain and the four mutant strains (Table 3), the OmpF‐deficient strain is less susceptible to the antibiotics compared to the wild type. The other mutant strains were either equally or more susceptible to the antibiotic. This IC_50_ data therefore indicates that OmpF is involved in the delivery of IMI, while the OmpC, OmpA, and OmpR proteins do not play an important role in facilitating the transport of this antibiotic across the outer membrane in *E. coli*.

**Table 3:** The experimental IC_50_ of IMI against *E. coli* Keio collection strains, reported in µg/mL.

Strain name	Strain trait	IC_50_ of IMI^a^ (µg/mL)
BW25113	Wild type	0.10
JW0912	Δ*ompF*	0.17
JW2203	Δ*ompC*	0.05
JW0940	Δ*ompA*	0.07
JW3368	Δ*ompR*	0.03

^a^ IC_50_ is defined as the concentration of antibiotics required to inhibit the growth of the bacteria by 50% compared to the untreated control.

**Figure 4:**
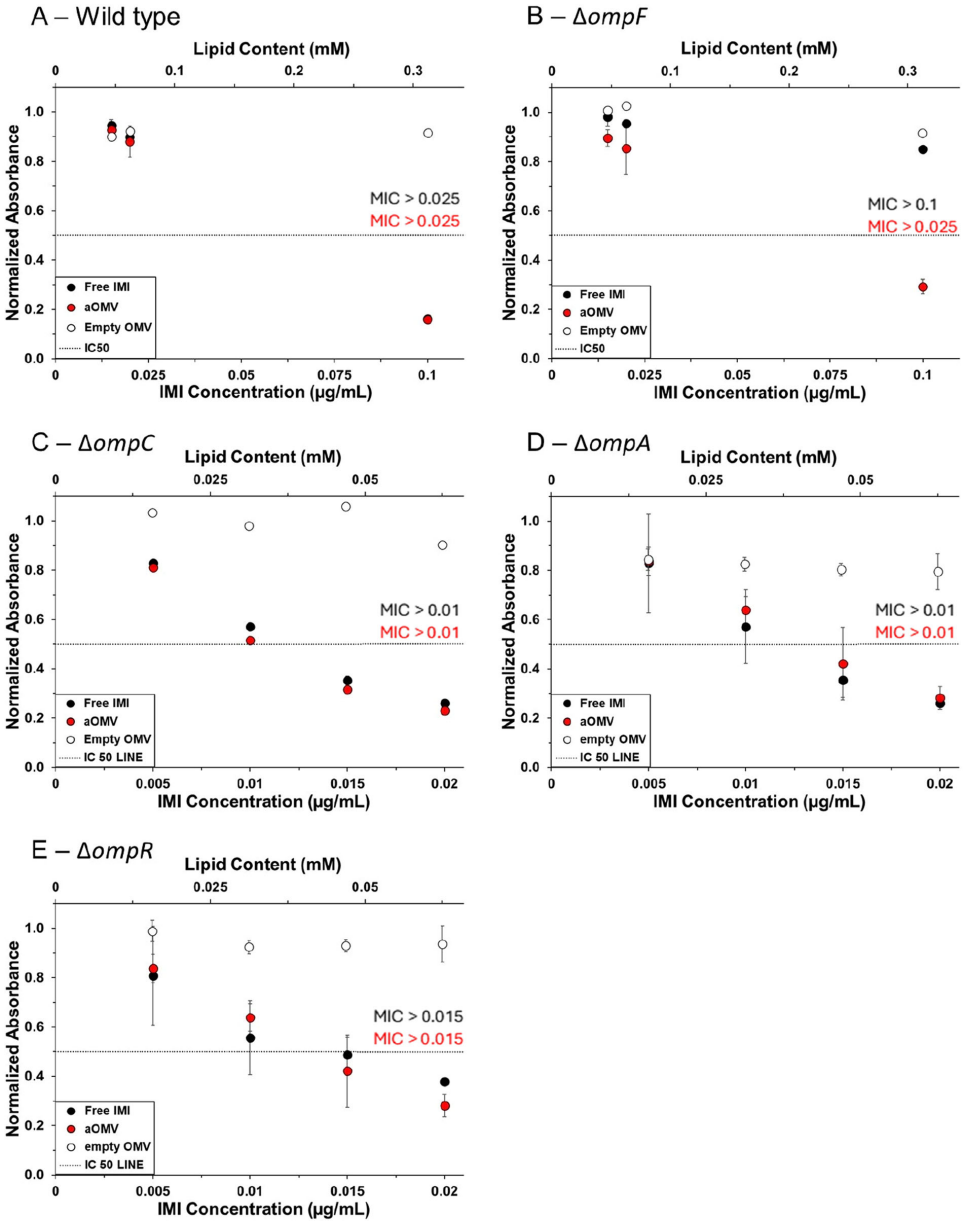
Treatment of *E. coli* Keio mutants with IMI. (A) BW25113 (wild type), (B) JW0912 (∆*ompF*), (C) JW2203 (∆*ompC*), (D) JW0940 (∆*ompA*), and (E) JW3368 (∆*ompR*). Each strain was treated with either free IMI (black), IMI‐OMVs (red), or empty OMVs (white). The change in absorbance at 600 nm after 12 h of growth of treated bacteria was normalized to that of untreated bacteria. The minimum inhibitory concentration (MIC) for free IMI and IMI‐OMVs is marked in black and red, respectively. The dashed line represents the IC_50_. Each data point represents the mean (*n* = 3) ± standard deviation.

To determine whether OMVs enable imipenem delivery in the absence of OmpF, we grew the wild type and mutant strains in the presence of (1) empty OMVs, (2) free IMI, or (3) IMI‐OMVs. In the absence of any treatment, all five strains grew in a similar manner (Figure S4). Both IMI‐OMVs and free IMI were equally effective in inhibiting the growth of the wild type strain (BW25113) at all concentrations (Figures 4A and S5). In contrast, IMI‐OMVs were significantly more effective than free IMI in treating JW0912 (∆*ompF*) (Figures 4B and S5, Table S3). Importantly, the growth of JW0912 in the presence of IMI‐OMVs was the same as that of the wild type strain treated with free IMI; encapsulation within OMVs restored the efficacy of IMI when OmpF was absent. Empty OMVs did not contribute to the growth inhibition. For the other strains, there was no significant difference between the antibacterial potency of IMI‐OMVs and free IMI, indicating that the absence of OmpC, OmpA or OmpR did not interfere with the internalization of IMI (Figures 4C–E and S5, Table S3). This result demonstrates that in the absence of OmpF, when free IMI is less effective due to its inability to cross the membrane, IMI‐OMVs are able enhance the effectiveness of IMI by promoting its uptake.

## 4 Discussion

In this study, we demonstrated that aOMVs can effectively inhibit the growth of Gram‐negative bacteria, including antibiotic‐resistant clinical isolates. Importantly, in most cases, encapsulation of the drugs within OMVs promoted the effectiveness of the antibiotics, as a lower concentration of antibiotics was needed to reach the same effect. While some OMVs have been reported to have bacteriolytic effects (Kadurugamuwa and Beveridge 1996), we showed that the *E.coli* JC8031 OMVs used in this study did not possess bacteriolytic activity. Thus, the increased effectiveness of the antibiotics can be attributed to enhanced delivery across the Gram‐negative membrane facilitated by the OMVs.

The outer membrane barrier limits the uptake of many antibiotics by Gram‐negative bacteria (Delcour 2009), with the pathway of drug uptake determined by the molecule's hydrophobicity and charge (Yang and Hinner 2015). Cationic aminoglycosides and polymyxins disrupt the outer membrane through electrostatic binding and gain access to the periplasmic space in the “self‐promoted uptake” pathway (Mohapatra et al. 2021; Nakae and Nakae 1982). Hydrophobic molecules can bind to the outer membrane and diffuse through the lipid bilayer (Pagès et al. 2008; Wardhan and Mudgal 2017), while small hydrophilic molecules can only gain entry by passing through nonspecific outer membrane proteins (Acosta‐Gutiérrez et al. 2018; Choi and Lee 2019; Pagès et al. 2008). When resistant bacteria modify the outer membrane permeability and downregulate the expression of outer membrane porin channels, such as OmpF, the uptake of small hydrophilic molecules is limited (Choi and Lee 2019; Novikova and Solovyeva 2009; Zhou et al. 2023), resulting in decreased effectiveness of these drugs.

Multiple studies have demonstrated that OMVs are able to deliver their cargo across this barrier. This phenomenon has frequently been demonstrated by showing that OMVs collected from antibiotic‐resistant organisms contain antibiotic resistance genes which can then be transferred to susceptible organisms. For example, clinical *Klebsiella pneumoniae* isolates were found to package the gene encoding for a specific carbapenemase (OXA‐232) into OMVs, which were able to deliver those genes to non‐resistant isolates (Shen et al. 2024). A similar mechanism was observed to describe the horizontal transfer of the gene encoding for the OXA‐24 β‐lactamase by *Acinetobacter baumannii* OMVs (Rumbo et al. 2011). Additionally, the hydrophobic nature of the OMV membrane enables the delivery of long‐chain hydrophobic quorum sensing molecules, such as N‐hexadecanoyl‐l‐homoserine lactone (C16‐HSL) produced by *Paracoccus denitrificans*, and 2‐heptyl‐3‐hydroxy‐4‐quinolone (pseudomonas quinolone signal, PQS), produced by *P. aeruginosa* to other bacteria (Mashburn and Whiteley 2005; Toyofuku et al. 2017). Although the detailed mechanisms of these OMV‐mediated transport processes have not yet been identified, this evidence indicates that OMVs are able to fuse with the outer membrane of Gram‐negative bacteria. Accordingly, Kadurugamuwa and Beveridge showed that after incubating *Salmonella typhimurium* or *E. coli* with OMVs produced by *Shigella flexneri* or *P. aeruginosa*, a significant amount of LPS from the donor bacteria could be detected on the surface of the acceptor bacteria, demonstrating the membrane mixing that occurs upon fusion (Kadurugamuwa and Beveridge 1999).

We therefore hypothesized that by encapsulating small hydrophilic antibiotics into OMVs, we could enable improved uptake of the antibiotics via fusion as a means of enhancing their activity. To investigate this hypothesis, we delivered IMI‐OMVs to an OmpF‐deficient mutant strain of *E. coli* and compared the resulting inhibition of growth against that of the wild type. We found that IMI‐OMVs were more effective against the OmpF deficient strain than unencapsulated antibiotics at the same concentration. The activity of IMI‐OMV in the OmpF knockout was similar to that of free drug in the wildtype strain. This finding supports our proposed mechanism by which aOMVs deliver luminal content via fusion with the bacterial outer membrane, thus delivering cargo in a porin‐independent manner. Our results build on the recent discovery that OMVs from a myxobacterium (SBSr073) were effective in delivering ciprofloxacin to the enteropathogen, *Shigella flexneri* (Heinrich et al. 2023). Thus, it appears that OMV‐mediated delivery of antibiotics could be a broad approach to improving antibiotic activity among various Gram‐negative bacterial species.

This observation that OMVs enable porin‐independent delivery of antibiotics opens several exciting possibilities, such as of broadening the spectrum of existing drugs. There are many more antibiotics that are effective against Gram‐positive bacteria than there are that are effective against Gram‐negative bacteria. In many cases, these molecules have such a narrow spectrum of activity, simply because their physical properties do not allow them to cross the Gram‐negative outer membrane (O'Shea and Moser 2008). For example, vancomycin, a tricyclic glycopeptide that irreversibly binds to d‐alanyl‐d‐alanine moieties in peptidoglycan to disrupt the bacterial cell wall, resulting in cell lysis (Sheldrick et al. 1978) is too large to cross the outer membrane or to pass through porins. As a result, it is ineffective against Gram‐negative bacteria because it is unable to access the peptidoglycan of these bacteria. We propose that OMVs could facilitate delivery of these types of molecules across the outer membrane, potentially increasing the effectiveness and broadening the spectrum of existing antibiotics, by enabling porin‐independent delivery.

## 5 Conclusions

In conclusion, this study demonstrates that aOMVs improve the activity of antibiotics by enhancing their transport across the Gram‐negative bacterial membrane. This porin‐independent delivery process enables improved activity of antibiotics, even when porin expression is decreased, and may be useful to allow delivery of large, narrow spectrum antibiotics to Gram‐negative bacteria. We demonstrated that this delivery method is effective in both lab strains and clinical antibiotic‐resistant isolates. Current work is focused on elucidating the mechanism of OMV‐outer membrane fusion to enable the design of biologically inspired synthetic systems with similar activity.

## Supporting information

Supplementary Information R1 clean.

## Data Availability

The data that support the findings of this study are available from the corresponding author upon reasonable request.
