# Supplementary material for: Porin‐Independent Uptake of Small Molecule Antibiotics Facilitated by Escherichia coli Outer Membrane Vesicles
Source: Biotechnol Bioeng. 2025 Oct 3;123(1):235–44. doi: 10.1002/bit.70078 (PMC12699110; doi:10.1002/bit.70078)
Supplement: Supplementary file 1 — Supplementary Information R1 clean. [file BIT-123-235-s001.docx]

**Porin-independent uptake of small molecule antibiotics facilitated by *Escherichia coli* outer membrane vesicles**

**Meishan Wu^1^, Rachael M. Harrower^2^, Ziang Li^1^, Angela C. Brown^1^**

**Supplementary Information**

^1^Department of Chemical and Biomolecular Engineering, Lehigh University, 124 E. Morton St., Bethlehem, PA, 18015, USA

^2^Department of Biological Sciences, Lehigh University, 111 Research Dr., Bethlehem, PA, 18015, USA


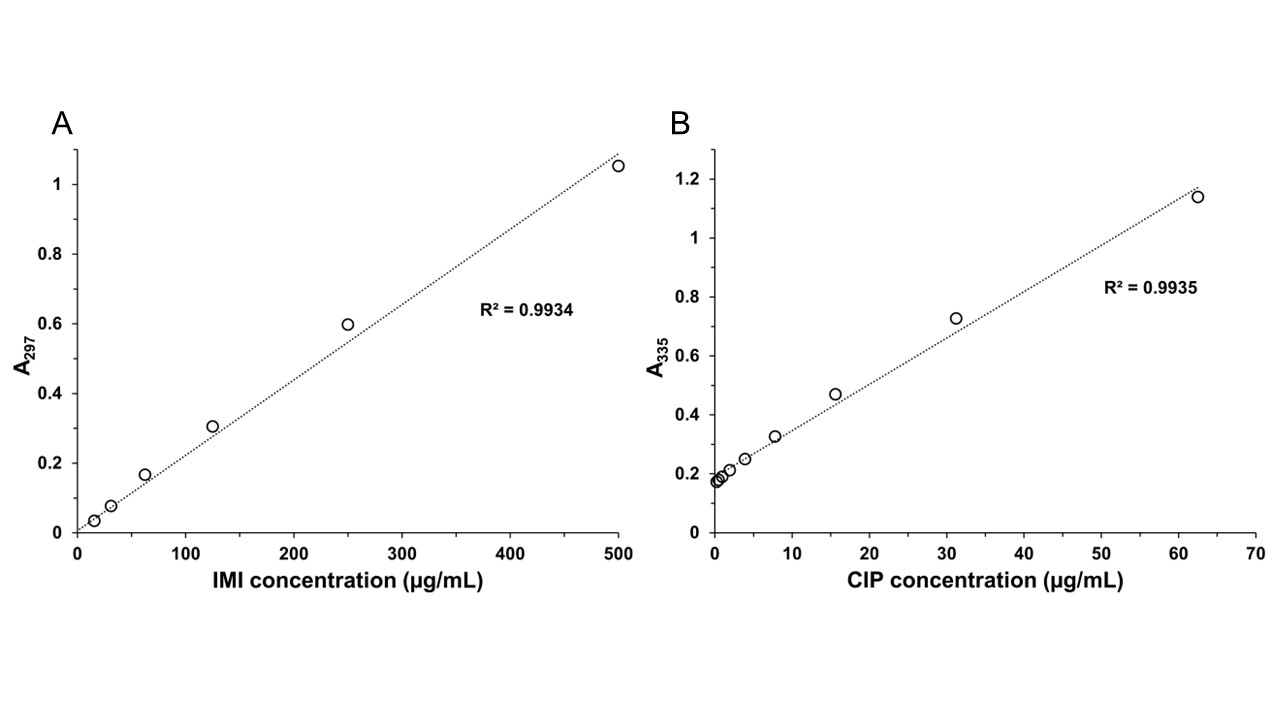


**Figure S1: Characteristic calibration curve for IMI used to determine the concentration of unloaded antibiotic.** The curve was fit using a linear regression, and the coefficient of determination (R^2^) is displayed.


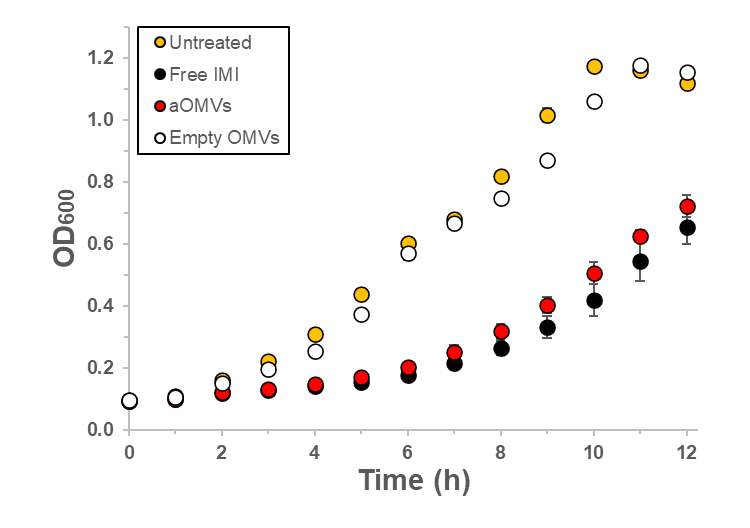


**Figure S2: Representative growth curves for *P. aeruginosa* PAO1.** The bacteria were untreated (yellow) or treated with free imipenem (black), aOMVs (red), or empty OMVs (white). The IMI concentration was 0.5 µg/mL. Each data point represents the mean (n=3) + standard deviation.

**Table S1: Statistical Analysis of PAO1 Data (Fig. 2)**

| **Concentration**  **(µg/mL)** | **p^1^** | **Significance^2^** | **% improvement^3^** |
| --- | --- | --- | --- |
|  |  |  |  |
| 0.5 | 0.1549 | NS | - |
| 1.0 | 0.8649 | NS | - |
| 2.0 | 0.2093 | NS | - |

^1^ Two-tailed p-value, determined using an unpaired t test, comparing the OD_600_ of bacteria treated with IMI-OMVs to those treated with free IMI.

^2^ NS, not significant

^3^ $\%improvement= \frac{\mathrm{OD}_{600}\left( free IMI \right)-\mathrm{OD}_{600}\left( IMI-OMVs \right)}{\mathrm{OD}_{600}\left( free IMI \right)}$

**C**


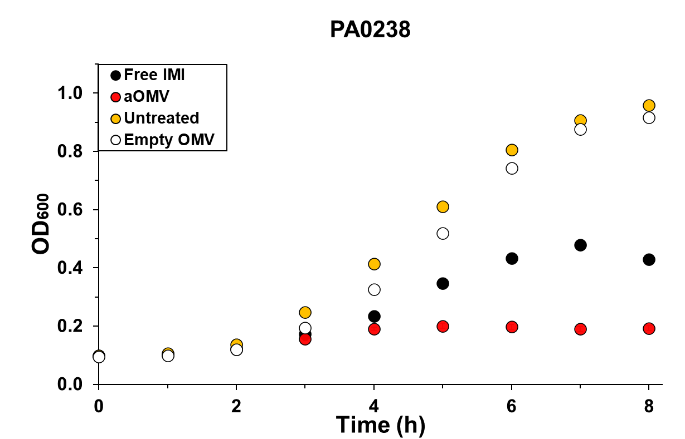

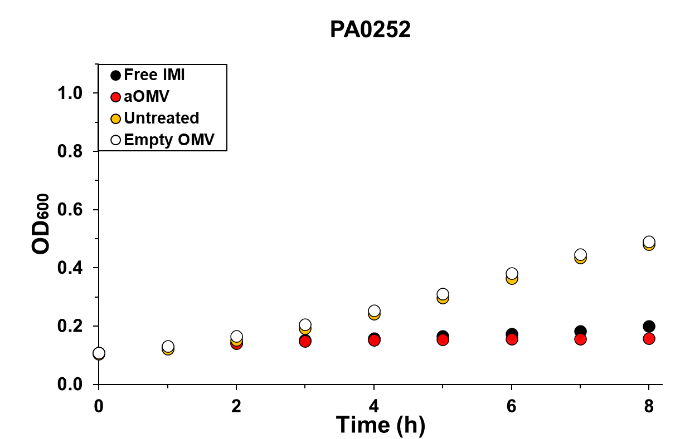

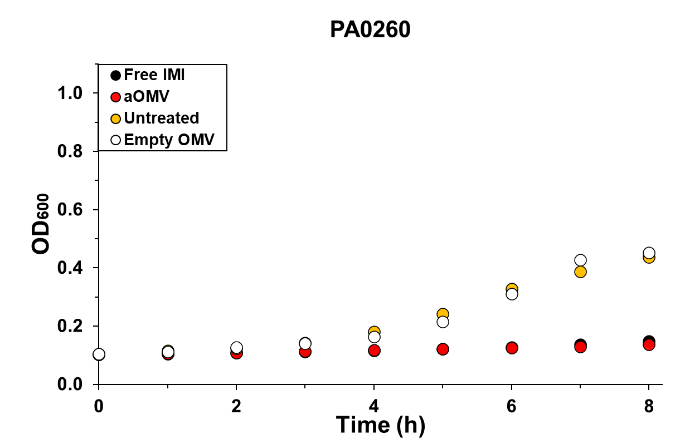


**A**

**B**

**Figure S3: Representative growth curves for (A) PA0238, (B) PA0252, and (C) PA0260.** The bacteria were untreated (yellow) or treated with free imipenem (black), aOMVs (red), or empty OMVs (white). PA0238 was treated with 0.1 µg/mL imipenem (free or in aOMVs), PA0252 was treated with 1 µg/mL imipenem (free or in aOMVs), and PA0260 was treated with 1.5 µg/mL imipenem (free or in aOMVs). Each data point represents the mean (n=3) + standard deviation.

**Table S2: Statistical Analysis of *P. aeruginosa* Clinical Isolates Data (Fig. 3)**

| **PA0238** | | | |
| --- | --- | --- | --- |
| **Concentration**  **(µg/mL)** | **p^1^** | **Significance^2^** | **% improvement^3^** |
| 0.05 | 0.0001 | *** | 22.2% |
| 0.10 | <0.0001 | *** | 48.3% |
| 0.15 | <0.0001 | *** | 35.8% |
| 0.20 | 1 | NS | - |
|  |  |  |  |
| **PA0252** | | | |
| **Concentration**  **(µg/mL)** | **p^1^** | **Significance^2^** | **% improvement^3^** |
| 0.50 | 0.275 | NS | - |
| 1.00 | 0.0003 | *** | 35.0% |
| 1.50 | 0.0213 | * | 4.9% |
| 2.00 | 0.0535 | NS | - |
|  |  |  |  |
| **PA0260** | | | |
| **Concentration**  **(µg/mL)** | **p^1^** | **Significance^2^** | **% improvement^3^** |
| 1.00 | 0.00002 | *** | - |
| 1.50 | 0.0010 | *** | 22.1% |
| 2.00 | 0.0030 | * | 15.1% |
| 2.50 | 0.0535 | NS | - |

^1^ Two-tailed p-value, determined using an unpaired t test, comparing the OD_600_ of bacteria treated with IMI-OMVs to those treated with free IMI

^2^ NS, not significant; *, p < 0.05; ***, p < 0.001

^3^ $\%improvement= \frac{\mathrm{OD}_{600}\left( free IMI \right)-\mathrm{OD}_{600}\left( IMI-OMVs \right)}{\mathrm{OD}_{600}\left( free IMI \right)}$

**
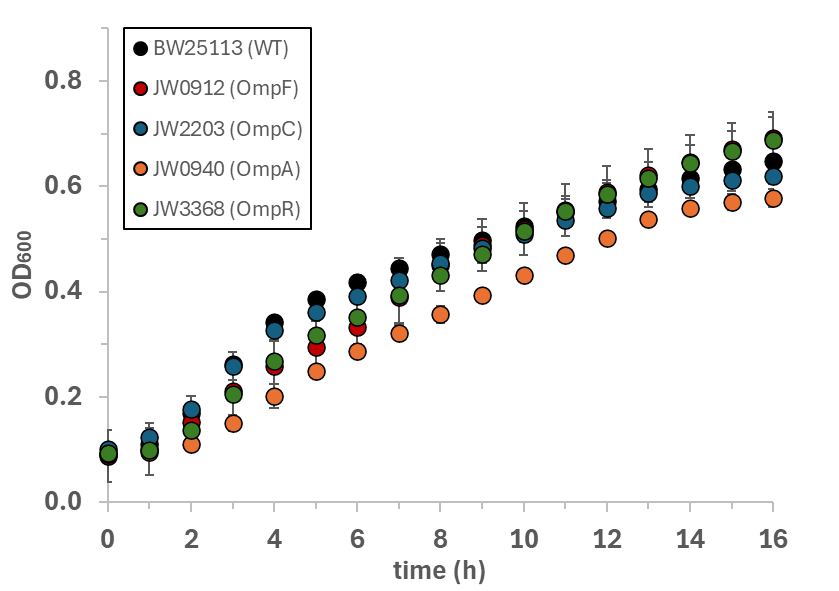
**

**Figure S4: Representative growth curves of Keio mutants.** The strains used in this work included BW25113 (wildtype, black), JS0912 (Δ*ompF*, red), JW2203 (Δ*ompC*, blue), JW0940 (Δ*ompA*, orange), and JW3368 (Δ*ompR*, green). All strains were grown in LB low salt broth, supplemented with 25 µg/mL of kanamycin. Each data point represents the mean (n=3) + standard deviation.


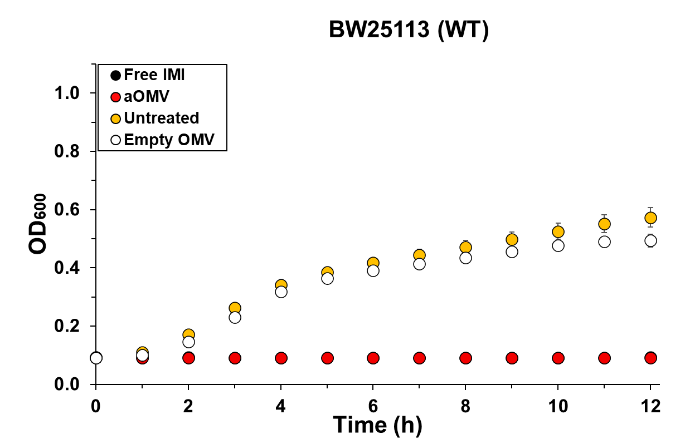


**A**


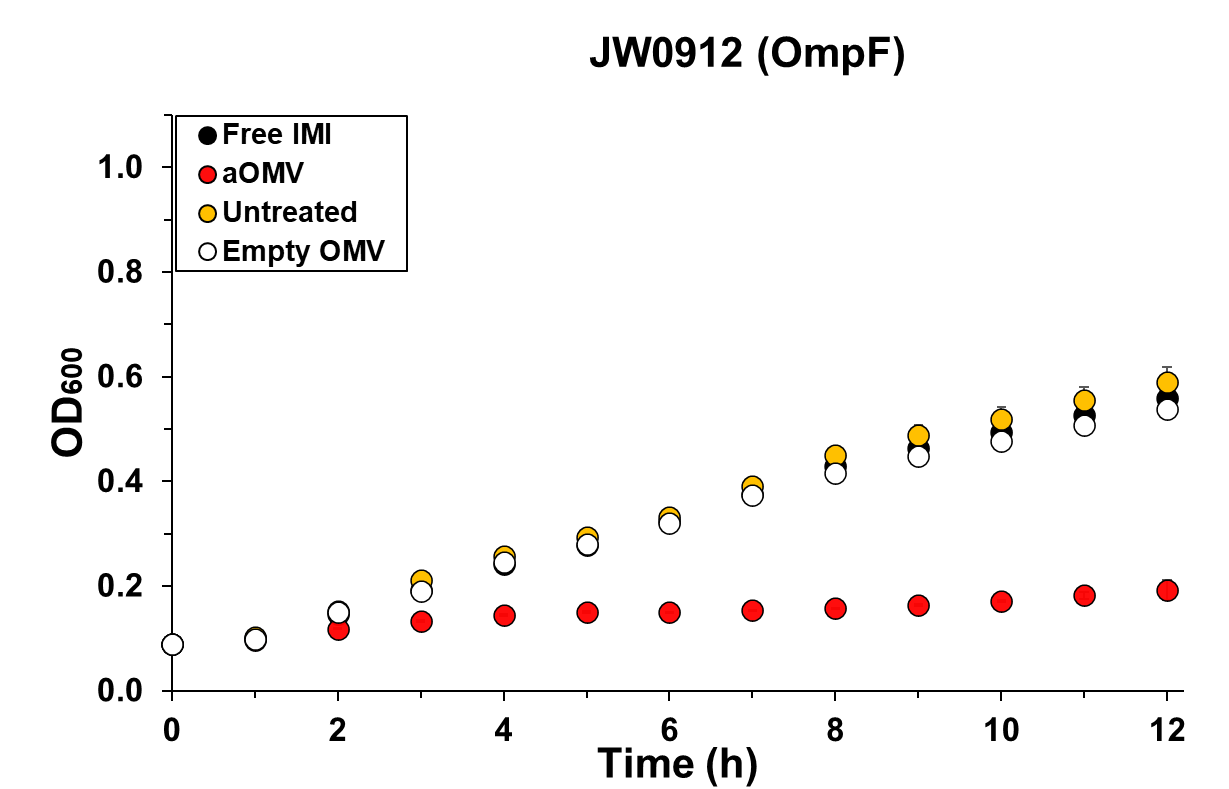


**B**


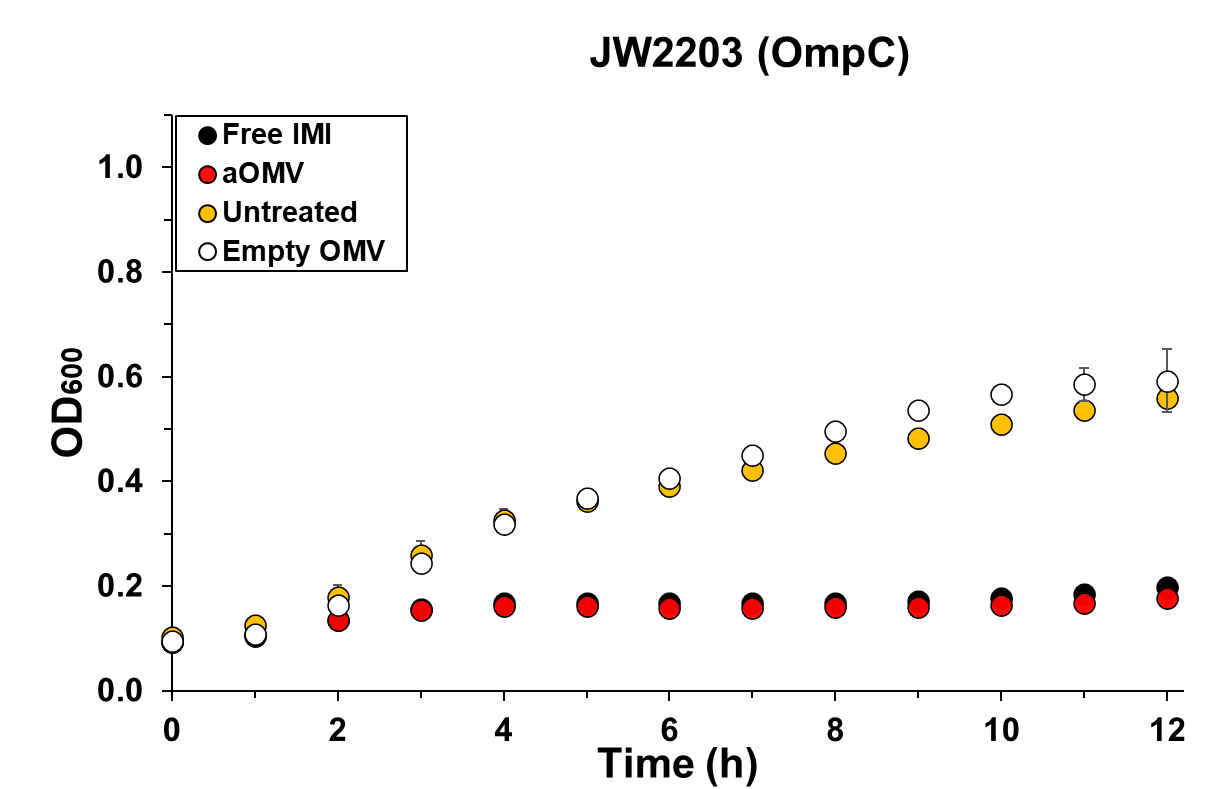


**C**


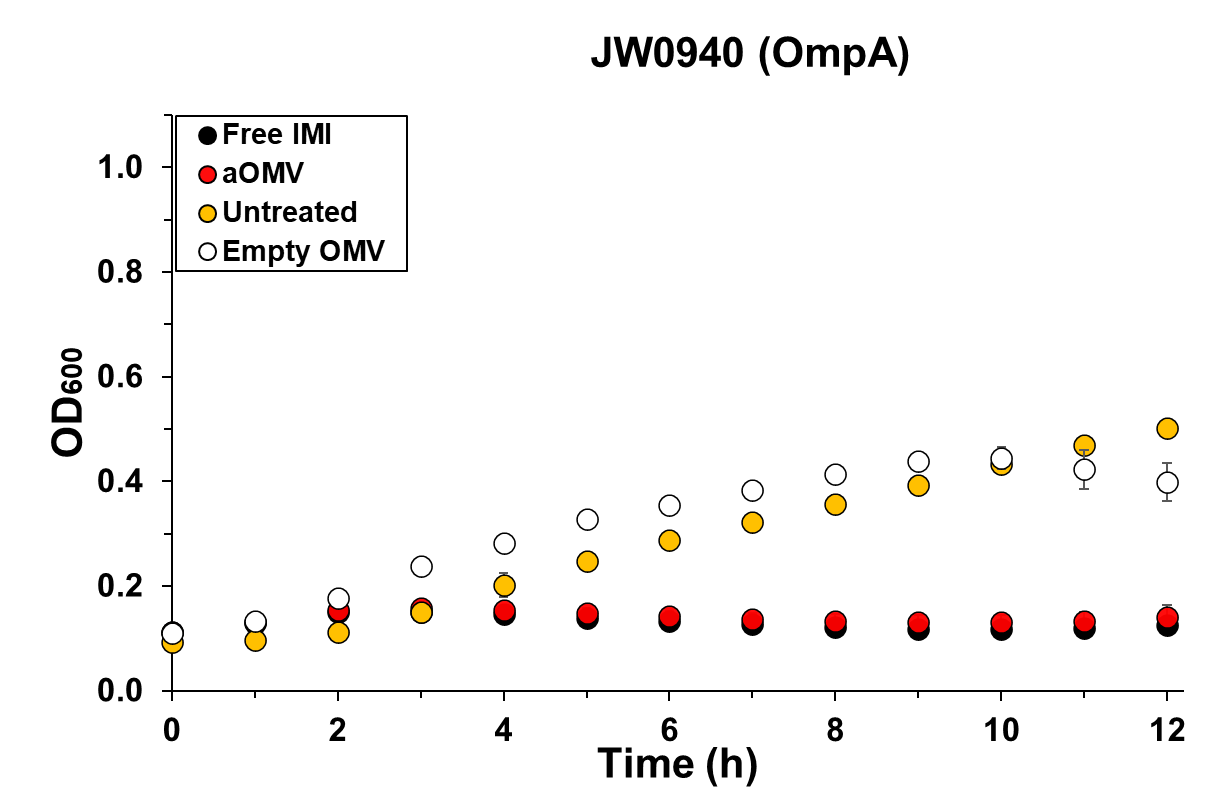


**D**


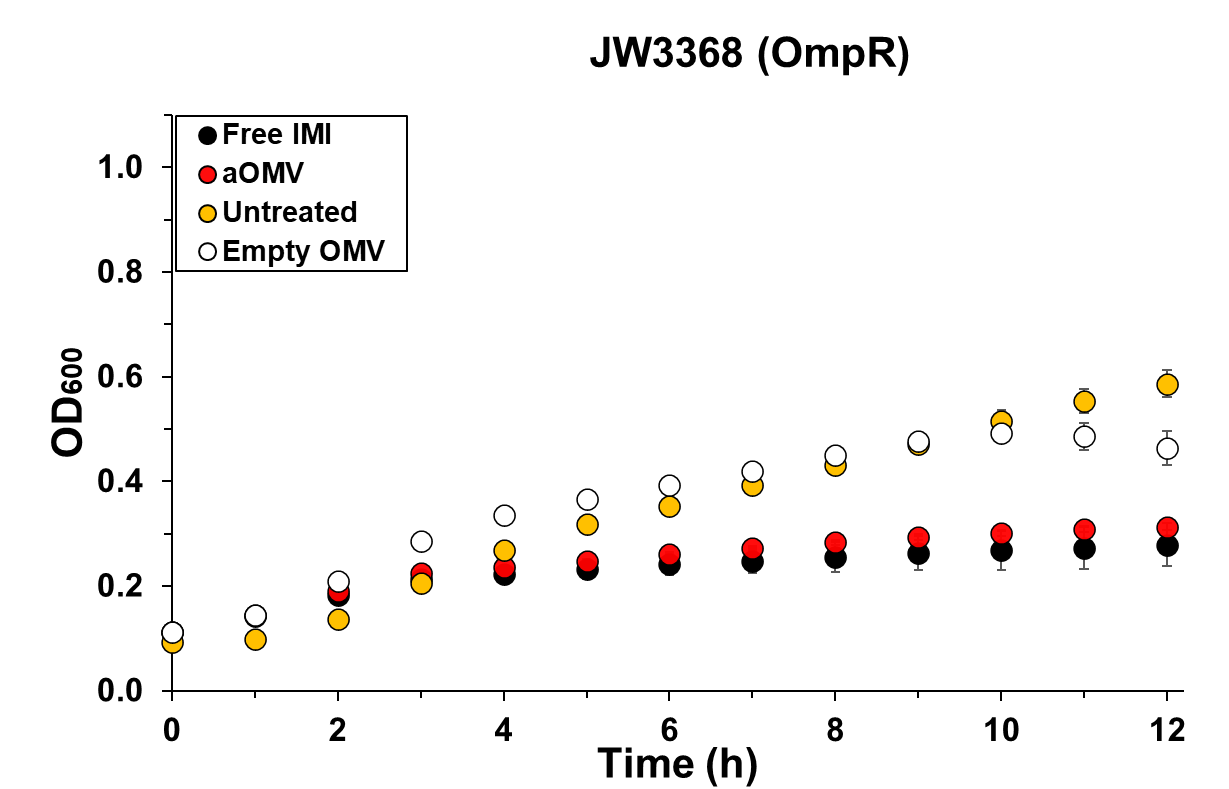


**E**

**Figure S5: Representative growth curves for Keio mutants treated with IMI.** (A) BW25113 (wildtype), (B) JW0912 (Δ*ompF*), (C) JW2203 (*ΔompC*), (D) JW0940 (*ΔompA*), and JW3368 (*ΔompR*). The bacteria were untreated (yellow) or treated with free IMI (0.01 µg/mL, black), aOMVs with an IMI concentration of 0.01 µg/mL (red), or empty OMVs with the same lipid concentration as the aOMVs (white). Each data point represents the mean (n=3) + standard deviation.

**Table S3: Statistical Analysis of Keio Mutants Data (Fig. 4)**

| **BW25113 (Wild type)** | | | |
| --- | --- | --- | --- |
| **Concentration**  **(µg/mL)** | **p^1^** | **Signficance^2^** | **%improvement^3^** |
| 0.01 | 0.0056 | ** | 5.9% |
| 0.02 | 0.6343 | NS | - |
| 0.10 | 0.511 | NS | - |
|  |  |  |  |
| **JW0912 (Δ*ompF*)** | | | |
| **Concentration**  **(µg/mL)** | **p^1^** | **Signficance^2^** | **%improvement^3^** |
| 0.01 | 0.911 | NS | - |
| 0.02 | 0.1724 | NS | - |
| 0.10 | <0.0001 | *** | 65.6% |
|  |  |  |  |
| **JW2203 (Δ*ompC*)** | | | |
| **Concentration**  **(µg/mL)** | **p^1^** | **Signficance^2^** | **%improvement^3^** |
| 0.005 | 0.1589 | NS | - |
| 0.010 | 0.0025 | ** | 9.8% |
| 0.015 | 0.0156 | NS | - |
| 0.020 | 0.0154 | NS | - |
|  |  |  |  |
| **JW0940 (Δ*ompA*)** | | | |
| **Concentration**  **(µg/mL)** | **p^1^** | **Signficance^2^** | **%improvement^3^** |
| 0.005 | 0.8029 | NS | - |
| 0.010 | 0.7526 | NS | - |
| 0.015 | 0.2837 | NS | - |
| 0.020 | 0.3404 | NS | - |
|  |  |  |  |
| **JW3368 (Δ*ompR*)** | | | |
| **Concentration**  **(µg/mL)** | **p^1^** | **Signficance^2^** | **%improvement^3^** |
| 0.005 | 0.2642 | NS | - |
| 0.010 | 0.2135 | NS | - |
| 0.015 | 0.0705 | NS | - |
| 0.020 | 0.8336 | NS | - |

^1^ Two-tailed p-value, determined using an unpaired t test, comparing the OD_600_ of bacteria treated with IMI-OMVs to those treated with free IMI

^2^ NS, not significant; *, p < 0.05; ***, p < 0.001

^3^ $\%improvement= \frac{\mathrm{OD}_{600}\left( free IMI \right)-\mathrm{OD}_{600}\left( IMI-OMVs \right)}{\mathrm{OD}_{600}\left( free IMI \right)}$
